# Performance Analysis of IEEE 802.11p MAC with Considering Capture Effect under Nakagami-*m* Fading Channel in VANETs

**DOI:** 10.3390/e25020218

**Published:** 2023-01-22

**Authors:** Yang Wang, Jianghong Shi, Lingyu Chen

**Affiliations:** School of Informatics, Xiamen University, Xiamen 361005, China

**Keywords:** vehicular ad hoc networks, medium access control, IEEE 802.11p, nakagami-*m* fading, markov model, capture effect, saturated throughput, average packet delay

## Abstract

Vehicular ad hoc networks (VANETs) have recently drawn a large amount of attention because of their enormous potential in road safety improvement and traffic management as well as infotainment service support. As the standard of medium access control (MAC) and physical (PHY) layers for VANETs, IEEE 802.11p has been proposed for more than a decade. Though performance analyses of IEEE 802.11p MAC have been performed, the existing analytical methods still need to be improved. In this paper, to assess the saturated throughput and the average packet delay of IEEE 802.11p MAC in VANETs, a two-dimensional (2-D) Markov model is introduced by considering the capture effect under Nakagami-*m* fading channel. Moreover, the closed-form expressions of successful transmission, collided transmission, saturated throughput, and average packet delay are carefully derived. Finally, the simulation results are demonstrated to verify the accuracy of the proposed analytical model, which also proves that this analytical model is more precise than the existing ones in terms of saturated throughput and average packet delay.

## 1. Introduction

In recent years, vehicular ad hoc networks (VANETs) have captured a great deal of attention from both industry and academia because of their enormous potential in improving road safety, efficiency of traffic management, and diversity of infotainment services [1,2]. To support both safety and non-safety applications, vehicle-to-vehicle (V2V), vehicle-to-pedestrian (V2P), vehicle-to-infrastructure (V2I), and vehicle-to-network (V2N) communication modes are all required in VANETs. However, accessing the wireless channel effectively is key to supporting differentiated applications [3]. As one of the main channel access standards for VANETs, IEEE 802.11p outlines the specifications of medium access control (MAC), i.e., the distributed coordination function (DCF) and the enhanced distributed channel access (EDCA). There are four access categories (ACs) defined in EDCA, and each AC queue, as an enhanced variant of the DCF, called an enhanced distributed channel access function (EDCAF) that contends for transmission opportunities (TXOPs) using a set of EDCA parameters [4]. It is worth mentioning that DCF and EDCA will still be adopted in the evolving version, i.e., IEEE 802.11bd [5]. Since the DCF protocol is the fundamental channel access mechanism, it is necessary to complete a precise performance analysis of IEEE 802.11p DCF in VANETs.

Though many analytical models have been proposed to analyze the DCF protocol, none of them are very precise, especially for those that do not consider the capture effect (i.e., the common phenomenon in wireless channels) [6,7,8,9,10,11,12,13,14,15,16,17,18,19]. In wireless communication scenarios, the capture effect may occur when the received signal power from one transmitter is higher than that of the others at the receiver. In actuality, the existence of the capture effect is beneficial to the system performance of the IEEE 802.11-based wireless networks [20,21,22]. However, only a few analytical models consider the capture effect under different fading channels in VANETs [20,21,22,23,24,25]. Since the Nakagami-*m* fading is more in line with the wireless channel environment in VANETs [26], it is necessary to analyze the IEEE 802.11p DCF under this fading channel model. Though the capture effect under the Nakagami-*m* fading channel is considered in [25], only the formulation for the normalized throughput is given, not the close-formed one. The average packet delay is also not mentioned, even though it is an important metric. To the best of our knowledge, there is no existing analytical model deriving the close-formed expressions of successful transmission, collided transmission, saturated throughput, and average packet delay together while also considering the capture effect under Nakagami-*m* fading channel in VANETs. Motivated by this, we propose a novel analytical model to evaluate the real performance of IEEE 802.11p DCF by different metrics in VANETs. The main contributions are twofold.

A 2-D Markov model is introduced and the close-formed expressions of successful transmission, collided transmission, saturated throughput and average packet delay while also considering the capture effect under Nakagami-*m* fading channel are carefully derived.

The simulation results are presented to verify the accuracy of the proposed analytical model in terms of saturated throughput and average packet delay, which has a more precise accuracy than existing models. In fact, the capture effect can increase the saturated throughput and lower the average packet delay.

## 2. Related Works

G. Bianchi first proposed a two-dimensional (2D) Markov chain model (i.e., the famous Bianchi’s model [6]) to analyze the saturation throughput of the DCF protocol, with the assumptions of no backoff freezing mechanism and no retry limits. Since then, a great deal of research has been conducted based on this pioneering work. For instance, the authors in [7] presented an extension of Bianchi’s model to a nonsaturated environment, while the authors in [8] particularly considered the backoff freezing mechanism. In [9], the authors modeled collision alleviating DCF with finite retry limits. In [10], the authors introduced two models for analyzing the access delay and throughput of DCF in a single-hop setting under both saturated and unsaturated traffic loads. In [11], the authors analyzed the saturated throughput and delay of the IEEE 802.11p unicast with one AC (i.e., DCF), and then proposed an optimization methodology to improve the performances of DCF. In [12], the broadcast performance of the IEEE 802.11 MAC is analyzed in VANETs under the nonsaturated condition based on a one-dimensional (1D) Markov model modified from Bianchi’s model. The authors in [13] also investigated the throughput stability of DCF networks, and the authors in [14] modelled the EDCA with considering freezing mechanism, finite retry limits, and idle state for four ACs under both saturated and nonsaturated conditions.

However, all of the above-mentioned studies are based on the hypothesis of ideal (a.k.a. perfect) channel condition. Hence, the authors in [15] analyzed the system performance of the DCF based on Bianchi’s model under different channel conditions. In addition, the authors in [16] presented a probabilistic analysis of the communication performance with the DCF in a multi-platooning scenario, while the transmission error for a packet is assumed as a constant probability. Similarly, the authors in [17] also adopted the fixed probability to represent the propagation error when analyzing the saturation throughput of steganography in the IEEE 802.11p DCF protocol. In [18], the authors also analyzed the IEEE 802.11p EDCA under saturation traffic condition with constant probabilities of transmission errors. In [19], the authors proposed an analytical model for NC-PNC MAC (a hybrid MAC based on the DCF) to investigate the performance of periodical broadcast of safety messages in VANETs under the Nakagami-*m* fading channel, where the erasure probability was 0 if a receiver was located within the communication range of a transmitter, and if not, the average erasure probability was used. Unfortunately, all of these studies ignore the influence from capture effect in their analytical models.

Since the capture effect is a common phenomenon in wireless networks, it must be considered for the analytical results to be accurate [20]. Moreover, the authors in [21] show that the capture effect can lead to a significant throughput gain in CSMA networks, where the wireless channel has undergone block Rayleigh fading. In [22], the authors analyzed the saturation throughput of DCF with heterogeneous node transmit power levels while also considering the capture effect under the free-space propagation model. The authors in [27] considered the capture effect under free-space propagation model but used it to analyze the broadcast performance of DCF. Moreover, the capture effect under free-space propagation model was also adopted in [28]. The authors in [23] analyzed the saturation throughput of the DCF by considering the capture effect in a Rayleigh fading environment; in [24], the authors also analyzed the saturation throughput of the DCF, but they considered both the capture effect in a Rayleigh fading channel as well as the retry limits. Moreover, the authors in [25] analyzed the DCF with the capture effect in Hoyt, Rice, and Nakagami-*m* fading environments, but the freezing mechanism and the retry limits were ignored and the authors only considered the system throughput without giving closed-form expressions.

Therefore, in this paper, a novel analytical model that considers the freezing mechanism, finite retry limits, and capture effect under Nakagami-*m* fading channel is proposed to analyze the performance of IEEE 802.11p DCF more accurately. And then, the closed-form expressions of successful transmission, collided transmission, saturated throughput, and average packet delay are carefully derived. It should be pointed out that, as the DCF is the basis of EDCA, this analytical model can be easily extended to analyze the EDCA in VANETs.

## 3. Analytical Model

### 3.1. Description of DCF

In DCF-based networks, the nodes contend for the channel by using a carrier sense multiple access with collision avoidance (CSMA/CA) with a slotted binary exponential backoff (BEB) scheme. According to the DCF, each node with a packet to transmit senses the channel first. If the channel is idle for a period of time that exceeds the distributed inter frame space (DIFS), the node transmits the packet. Otherwise, it defers the transmission to avoid collision by starting a random backoff process, where the transmission is only permitted at the beginning of slots. When the backoff is initiated, the random backoff time is uniformly chosen in the range [0, CW−1], where CW is the contention window with the minimum $W_{0}$ at the first transmission attempt. The backoff counter is decremented by one at the end of each idle slot. When the backoff counter reaches zero, the node commences transmission irrespective of the channel status. However, the counter is frozen when the channel is busy and resumed when the channel is idle again for more than a DIFS. If a packet (DATA) is successfully received, the receiver sends back an acknowledgement (ACK) after a period of short inter-frame space (SIFS) to inform the transmitter for the correct reception. If no ACK is received within a specified ACK timeout interval, the transmitter will assume a failed transmission and schedule a retransmission by BEB algorithm. That is, CW is doubled and another backoff period is initiated. If the maximum of contention window ($W_{M}=2^{M}W_{0}$) is reached and the transmission is still unsuccessful, CW is maintained at $W_{M}$ for at most f attempts. Otherwise, the packet will be discarded. The CW is reset to $W_{0}$ after a successful transmission or reaching the packet’s retry limits (M+f).

In fact, the DCF includes two access techniques: the basic access mode and the request-to-send/clear-to-send (RTS/CTS) mode. The former detailed above is a default two-way handshaking mechanism using DATA/ACK packets and the latter is a four-way handshaking mechanism using RTS/CTS packets to reserve the channel before packet transmission. The RTS/CTS mode follows the same backoff rules as the basic mode, which is to reduce the risk of large packet collision caused by the hidden node problem. As these two control packets include the duration of the ongoing transmission, the nodes update their network allocation vector (NAV) by the received RTS or CTS and defer the transmission for the specified duration to avoid collision. The major notions used in the analysis model are summarized in Table 1.

### 3.2. Probabilities of Transmission and Collision

According to [25], when the received signal power which comes from one node is greater than the sum of that of the others, the capture effect occurs at the targeted node. Since the Nakagami-*m* fading is more in line with the characteristics of the wireless channel in VANET scenario, it has been widely adopted in the performance analysis of VANETs [26]. Therefore, in this paper, the performance analysis of the DCF is under the Nakagami-*m* fading channel with considering the capture effect. For an inference-limited system, the condition for capture is $\gamma_{t}/\overset{n}{\underset{k=1,k\neq t}{\sum }}\gamma_{k}>z$, where $\gamma_{t}$ is concerned signal power from one node, $\;\gamma_{k}$ is the interference signal power from other nodes, and z  is the capture threshold. Under the hypothesis of perfect power control, the capture probability conditioned on n−1 interferers for the concerned node can be calculated by:

(1) $$\begin{array}{ll} p_{cap}(n,z) & =\int_{0}^{\infty }f_{\gamma_{t}}(\gamma_{t})\cdot \mathrm{Pr}\left[\gamma_{t}/\sum_{k=1,k\neq t}^{n}\gamma_{k}>z\right]d\gamma_{t}\\ & =\int_{0}^{\infty }f_{\gamma_{t}}(\gamma_{t})\left[\int_{0}^{\gamma_{t}/z}f_{\gamma_{n-1}}(\gamma_{n-1})d\gamma_{n-1}\right]d\gamma_{t}\\ & \underline{\underline{\left(a\right)}}\frac{1}{\Gamma (m)\Gamma (mn-m)}\sum_{k=0}^{\infty }\frac{{\left(-1\right)}^{k}\Gamma (mn+k)}{k!(mn-m+k)z^{mn-m+k}} \end{array}$$

where $f_{\gamma_{t}}\left(\gamma_{t}\right)$ is the instantaneous received power in Nakagami-*m* fading channel and $f_{\gamma_{n-1}}\left(\gamma_{n-1}\right)$ is the n−1 fold convolution of $f_{\gamma_{t}}\left(\gamma_{t}\right)$, which are respectively expressed as:

(2) $$f_{\gamma_{t}}(\gamma_{t})=\frac{m^{m}\gamma_{t}{}^{m-1}}{{\bar{\gamma }}^{m}\Gamma (m)}e^{-\frac{m\gamma_{t}}{\bar{\gamma }}},\gamma \geq 0$$

(3) $$f_{\gamma_{n-1}}(\gamma_{n-1})=\frac{m^{m(n-1)}}{{\bar{\gamma }}_{}^{m(n-1)}\Gamma (mn-m)}\gamma_{n-1}^{m(n-1)-1}e^{-\frac{m\gamma_{n-1}}{\bar{\gamma }}}$$

where m is the shape parameter ranging from 1/2 to ∞. $\bar{\gamma }$ is the average received power determined by $\bar{\gamma }=P_{tx}\cdot C\cdot r_{i}^{-\alpha }$, where $P_{tx}$ is the transmission power, α is the path-loss exponent, and C is a constant related to the antenna gains, the carrier frequency, and the speed of light, which are the same for all nodes. For simplicity, the average received power from different nodes is the same for the receiver [21,29,30]. In fact, if the uplink is considered in VANETs, the RSU only cares about the number of vehicles in its coverage. However, the distance between the vehicle and the RSU has an effect on the communication quality, which can lead to unfairness for different vehicles communicating with the RSU from different distances. Therefore, for fairness, the assumption of power control is made, which means that the average values of received power from different vehicles at the RSU are the same. This assumption is reasonable and can also be found in the references [21,23,24,29,31]. The derivation of step (*a*) in (1) is detailed in our previous work in [31].

**Figure 1:**
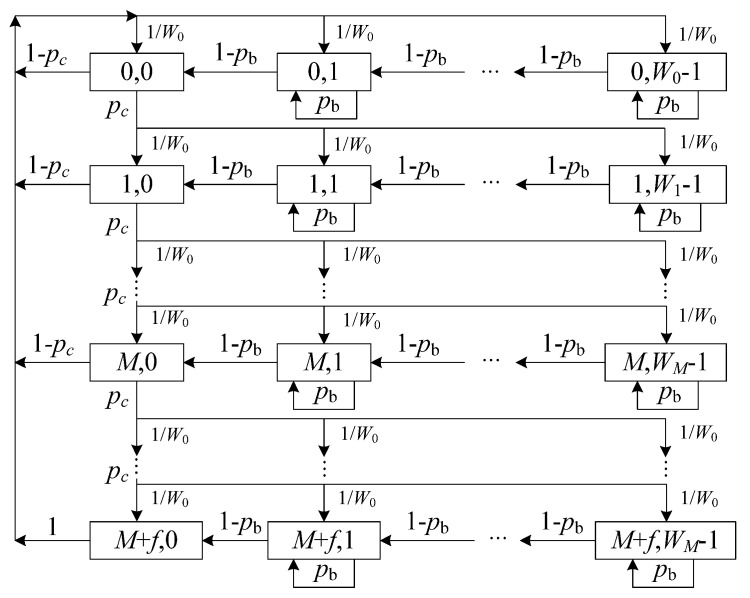
2-D Markov chain model.

As shown in Figure 1, a 2-D Markov chain model is introduced for modeling the behavior of the DCF protocol while considering finite retry limits and backoff counter freezing. In this model, the saturation condition is considered, i.e., each node always has at least one packet to transmit. Here, the states of a node at time t can be represented as {s(t), b(t)}, where s(t) with values from {0, 1,…, M+f} and b(t) with values from {0, 1,…, $W_{i}-1$}) are defined as the random backoff stage and the value of backoff counter at time t, respectively. These random variables are dependent because the maximum value of the backoff counter depends on the backoff stage:

(4) $$W_{i}=\{\begin{array}{c} 2^{i}W_{0},\;\;\;\;\;\;0\leq i\leq M\\ W_{M},\begin{array}{cc} & M<i\leq M+f \end{array} \end{array}$$

Let b(i,k) be the stationary distribution of the proposed 2-D Markov chains. Then, the one-step state transition probabilities can be given by:

(5a) $$P\left\{(i,k)|\left(i,k+1\right)\right\}=1-p_{b},k\in [0,W_{i}-1),i\in [0,M+f]$$

(5b) $$P\left\{(i,k)|\left(i,k\right)\right\}=p_{b},k\in (0,W_{i}-1],i\in [0,M+f]$$

(5c) $$P\left\{(i,k)|\left(i-1,0\right)\right\}=\frac{p_{c}}{W_{i}},k\in [0,W_{i}-1],i\in (0,M+f]$$

(5d) $$P\left\{(0,k)|\left(i,0\right)\right\}=\frac{1-p_{c}}{W_{0}},k\in [0,W_{i}-1],i\in [0,M+f)$$

(5e) $$P\left\{(0,k)|\left(M+f,0\right)\right\}=\frac{1}{W_{0}},k\in [0,W_{0}-1]$$

Therefore, we calculate the following steady state probabilities:

(6) $$b(i,0)={\left(p_{c}\right)}^{i}\cdot b(0,0),i\in (0,M+f]$$

(7) $$\begin{array}{ll} b(i,k) & =\frac{1}{1-p_{b}}\cdot \frac{W_{i}-k}{W_{i}}\cdot b(i,0)\\ & =\frac{W_{i}-k}{W_{i}}\cdot \frac{{\left(p_{c}\right)}^{i}}{1-p_{b}}\cdot b(0,0),k\in (0,W_{0}-1],i\in (0,M+f] \end{array}$$

Then, according to the normalization condition for stationary distribution, we have:

(8) $$1=\sum_{i=0}^{M+f}\sum_{k=0}^{W_{i}-1}b(i,k)$$

Therefore, based on (6)–(8), we can obtain:

(9) $$b(0,0)=\frac{2(1-p_{b})(1-p_{c})(1-2p_{c})}{\psi +2(1-p_{b})(1-2p_{c})(1-{\left(p_{c}\right)}^{M+f+1})}$$

where ψ is represented as:

(10) $$\begin{array}{cc} \psi = & W_{0}(1-p_{c})(1-{\left(2p_{c}\right)}^{M+1})-(1-2p_{c})(1-{\left(p_{c}\right)}^{M+f+1})\\ & +W_{0}p_{c}{\left(2p_{c}\right)}^{M}(1-2p_{c})(1-{\left(p_{c}\right)}^{f}) \end{array}$$

Therefore, the probability that a node transmits in a randomly chosen slot can be expressed as:

(11) $$\tau_{tra}=\sum_{i=0}^{M+f}b(i,0)=\frac{2(1-p_{b})(1-2p_{c})(1-{\left(p_{c}\right)}^{M+f+1})}{\psi +2(1-p_{b})(1-2p_{c})(1-{\left(p_{c}\right)}^{M+f+1})}$$

where $p_{b}$, the probability that the channel is busy for a concerned node, can be calculated as:

(12) $$p_{b}=1-{\left(1-\tau_{tra}\right)}^{n-1}$$

Since the capture effect is considered, the probabilities of collided transmission in a given slot for a concerned node can be calculated by:

(13) $$p_{c}=\sum_{j=1}^{n-1}\left[1-p_{cap}(j+1,z)\right]C_{n-1}^{j}{\left(\tau_{tra}\right)}^{j}{\left(1-\tau_{tra}\right)}^{n-j-1}$$

where $C_{n-1}^{i}=\left(n-1\right)!/\left[j!\left(n-j-1\right)!\right]$, and $\;p_{cap}\;\left(\cdot ,\cdot \right)$ is the capture probability calculated by (1). As seen, the nonlinear system composed of Equations (11) and (13) contains only two unknown parameters, i.e., $\tau_{tra}$ and $p_{c}$. Therefore, their values can be obtained by numerical calculation, and the system has a unique solution.

### 3.3. Normalized Throughput

According to [6], the average length of a virtual slot can be calculated as:

(14) $$E[Slot]=(1-p_{tra})\sigma +p_{s}^{}p_{tra}T_{s}+p_{tra}(1-p_{s}^{})T_{c}$$

Let η denote the normalized throughput, i.e., the ratio of the duration of successful transmission of the payload to the average length of a virtual slot, then we have:

(15) $$\eta =\frac{T_{s}^{E[PL]}}{E[Slot]}=\frac{p_{s}^{}p_{tra}E[PL]/R_{t}}{(1-p_{tra})\sigma +p_{s}^{}p_{tra}T_{s}+p_{tra}(1-p_{s}^{})T_{c}}$$

where E[PL] is the average payload of packet and $R_{t}$ is the channel rate. For simplicity, suppose all nodes send packets with the same length. $p_{tra}$ denotes the probability that at least one node transmits in a concerned slot, and $p_{s}$ denotes the probability that there is a node successfully transmits in a certain slot conditioned on that at least one node transmits. σ, $T_{s}$, $T_{c}$ denote the average durations of empty slot, successful transmission, and channel collision, respectively. Assume there are n nodes competing for the transmission chance in the network, then $p_{tra}$ can be computed by:

(16) $$p_{tra}=1-{\left(1-\tau_{tra}\right)}^{n}$$

Therefore $p_{s}$ can be calculated by:

(17) $$p_{s}^{}=\frac{p_{suc}^{}}{p_{tra}}=\frac{\sum_{i=1}^{n}C_{n}^{i}{\left(\tau_{tra}\right)}^{i}{\left(1-\tau_{tra}\right)}^{n-i}p_{cap}(i,z)}{1-{\left(1-\tau_{tra}\right)}^{n}}$$

where $p_{suc}$ is the probability of successful transmission in a randomly chosen slot and $C_{n}^{i}=n!/\left[i!\left(n-i\right)!\right]$.

For the basic mode, the average durations of successful transmission and failed transmission are computed as follows:

(18) $$\{\begin{array}{c} T_{s}^{bas}=T_{H}+T_{E[PL]}+T_{SIFS}+T_{PD}+T_{ACK}+T_{DIFS}+T_{PD}\\ T_{c}^{bas}=T_{H}+T_{E[PL]}+T_{DIFS}+T_{PD} \end{array}$$

where the packet header is $H=PHY_{hdr}+MAC_{hdr}$ and $T_{H}=\frac{H}{R_{t}}$ is the duration of transmitting it.$\;T_{E\left[PL\right]}=\frac{E\left[PL\right]}{R_{t}}$,$\;T_{SIFS}$,$\;T_{DIFS}$, and $T_{ACK}=\frac{ACK}{R_{t}}$ are the durations of transmitting the packet payload, SIFS, DIFS, and transmitting ACK, respectively. $T_{PD}$ is the propagation delay. For the RTS/CTS mode, we have:

(19) $$\{\begin{array}{c} T_{s}^{rts}=T_{RTS}+T_{SIFS}+T_{PD}+T_{CTS}+T_{SIFS}+T_{PD}+T_{H}+T_{E[PL]}+T_{SIFS}+T_{PD}+T_{ACK}+T_{DIFS}+T_{PD}\\ T_{c}^{rts}=T_{RTS}+T_{DIFS}+T_{PD} \end{array}$$

where $T_{RTS}=\frac{RTS}{R_{t}}$ and $T_{CTS}=\frac{CTS}{R_{t}}$ is the time duration required to transmit RTS and CTS frames, respectively.

### 3.4. Average Packet Delay

The average delay for a successfully transmitted packet is defined as the time interval from the beginning that the packet is at the head of MAC queue for transmission to the end that an ACK for this packet is successfully received. It should be pointed out that the delay time for a packet dropped due to reaching the retry limit is not included. Therefore, the average delay for a successful transmission can be calculated as:

(20) E[D]=E[Slot]⋅E[SlotNum]

where E[Slot] is the average length of a virtual slot represented as (14) and E[SlotNum] is the average number of virtual slots required for a successful transmission calculated by:

(21) $$E[SlotNum]=\frac{1}{\sum_{j=0}^{n-1}C_{n-1}^{j}{\left(\tau_{tra}\right)}^{j+1}{\left(1-\tau_{tra}\right)}^{n-j-1}p_{cap}(j+1,z)}-\frac{p_{drop}}{1-p_{drop}}\cdot E[X_{drop}]$$

where the first term is the expected number of virtual slots for the successfully transmitted packet (including the average duration of the dropped packet due to reaching the retry limit) and the second term is the average number of virtual slots of dropped packet due to reaching the retry limit conditioned on the existing successful transmission. The probability of a dropped packet being caused by reaching the retry limit ($p_{drop}$) and the average number of virtual slots required for a dropped packet ($E\left[X_{drop}\right]$) can be severally calculated by:

(22) $$p_{drop}={\left(p_{c}\right)}^{M+f+1}$$

(23) $$E[X_{drop}]=\sum_{i=0}^{M+f}\frac{W_{i}-1}{2}=\frac{W_{0}(2^{M+1}-1)+f\cdot W_{0}\cdot 2^{M}-M-f-1}{2}$$

Then, substitute (14), (21)–(23) and (18) (or (19)) into (20) with the values of $\tau_{tra}$ and $p_{c}$, the average packet delay of a successful transmission for the basic mode (or RTS/CTS mode) can easily be obtained.

## 4. Model Validation

**Figure 2:**
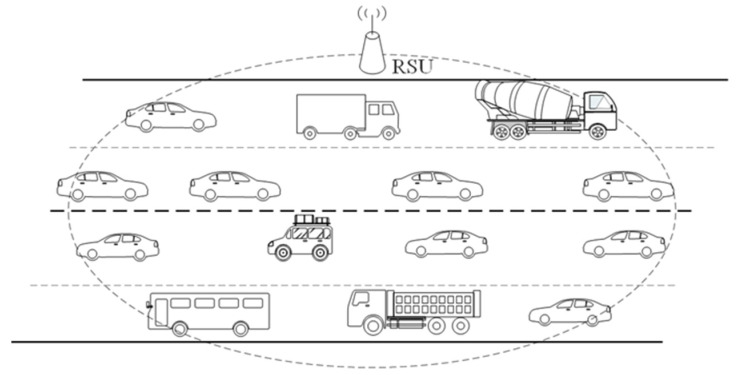
V2I scenario.

In this section, the simulation results are provided to validate the effectiveness of the proposed analytical model for both basic and RTS/CTS modes. All the results are calculated using MATLAB (as in references [3,21,26,27,31]) and the Monte Carlo method is used. The analytical results are calculated by the derived expressions, while the simulation results are found using an event-driven custom simulation program, written in MATLAB, that closely follows all the 802.11p DCF protocol details for each independently transmitting vehicle. For the sake of simplicity, the considered simulation scenario is that all vehicles communicate with a RSU as shown in Figure 2 (e.g., the drive-thru network scenario in [32]). In fact, the vehicle velocity, the traffic density, and the coverage of the RSU eventually lead to different numbers of vehicles in the coverage of the RSU. Therefore, we take the number of vehicles (which is equivalent to the vehicle density when the coverage of the RSU is given) into account for simplicity in the simulation, as in references [11,12,14,17,18,19,23,26,27,28,31].

To verify the improvement of the proposed model, it was compared with Cao’s model [14] with a single saturated AC queue (an extreme case) that uses the same simulation parameters for fairness. The main parameters used are listed in Table 2.

**Figure 3:**
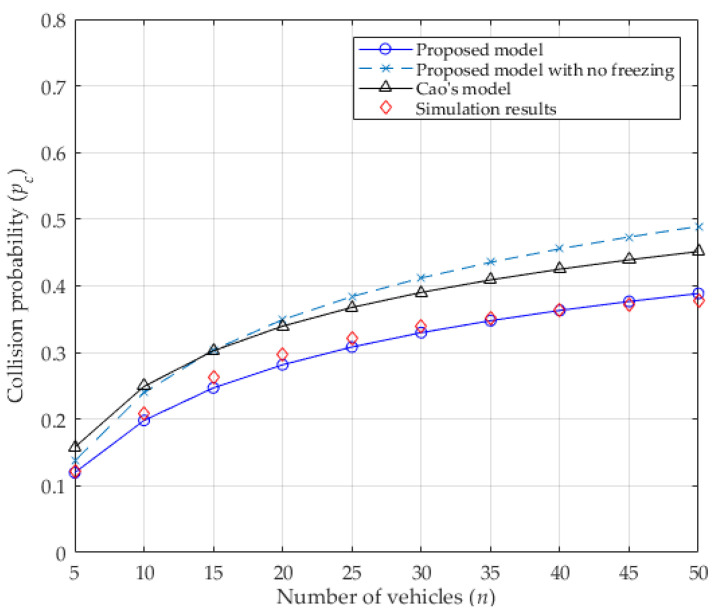
Collision probability.

**Figure 4:**
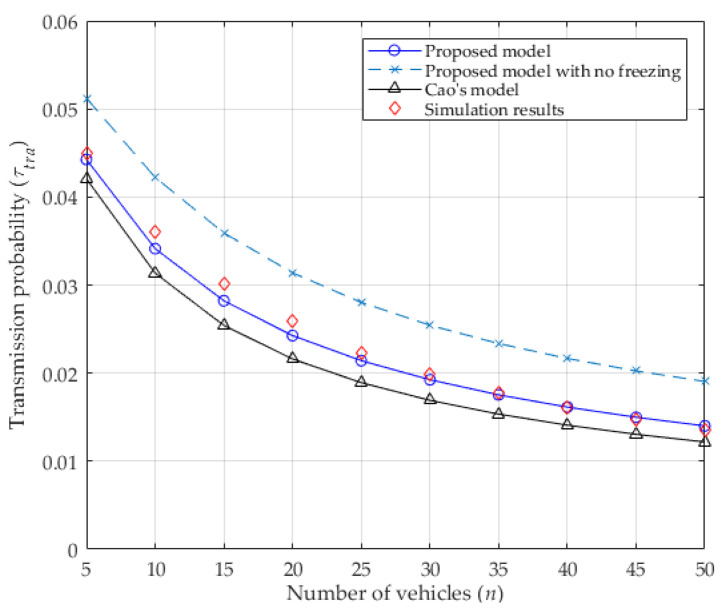
Transmission probability.

The transmission probability of a vehicle in a concerned slot depends on the values of $W_{0}$, M, f, and the number of contending vehicles n. It is also influenced by the collision probability from Equations (11) and (14). As seen in Figure 3, when the number of vehicles increases, the collision probability increases because more vehicles contend for the channel, which leads to more vehicles performing the backoff procedure and deferring from accessing the channel. This causes the transmission probability to decrease, as shown in Figure 4. In addition, the values of both collision probability and transmission probability of the proposed analytical model with no freezing mechanism are presented, which are all higher than that of considering it. Without the freezing mechanism, regardless of the channel’s condition, the backoff counter of the vehicle will always decrease by 1, as in [24] (inconsistent with the DCF protocol). That is to say, the vehicle can acquire the transmission opportunity with less waiting time, which enlarges the transmission probability of the vehicle. Accordingly, the collision probability will increase because more vehicles may transmit at the same time. Obviously, the values of both the collision probability and the transmission probability for the proposed analytical model are very close to the simulation results, meaning that they are more accurate than the values in Cao’s model. In other word, it is obvious that the capture effect can effectively decrease the collision probability and gently increase the transmission probability. Therefore, the capture effect must be considered when analyzing the IEEE 802.11p DCF protocol in VANETs.

**Figure 5:**
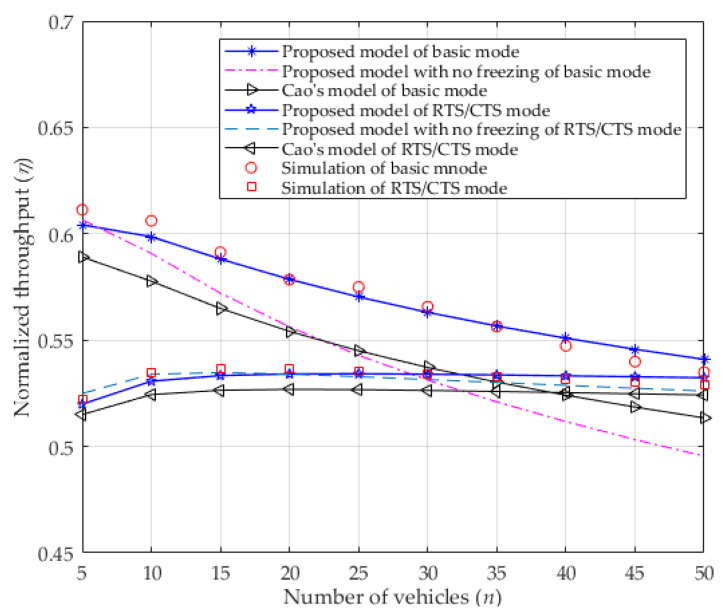
Normalized throughput.

In Figure 5, the normalized saturated throughput is given for both basic mode and RTS/CTS mode. Obviously, the saturated throughput decreases as the number of contending vehicles increases in the basic mode because more vehicles contend for the channel, leading to wasted channel resources. In the RTS/CTS mode, even though more vehicles want to access the channel, the usage of RTS/CTS can decrease the packet collision, which eventually stabilizes the normalized throughput. Since ignoring the freezing mechanism in the proposed model enlarges the collision probability (shown in Figure 3), the normalized throughput is lower than that of considering it, especially in the basic mode. This occurs because more collisions result in more waste of channel resources, which will lower the normalized throughput. At the same time, the analytical results of the proposed model are very consistent with the simulation results, which are all higher than the results of Cao’s model, which does not consider the capture effect. In fact, the normalized saturated throughput can be improved distinctly by the capture effect, especially in the basic mode. That is to say, the basic mode is more sensitive to the capture effect than the RTS/CTS mode. This occurs because the capture effect can make transmission more successful. In addition, when the number of vehicles is increasing, the saturated throughput of the RTS/CTS mode can be higher than that of the basic mode for the situation when the packet collision is replaced by a smaller length of colliding packets (i.e., RTS/CTS).

**Figure 6:**
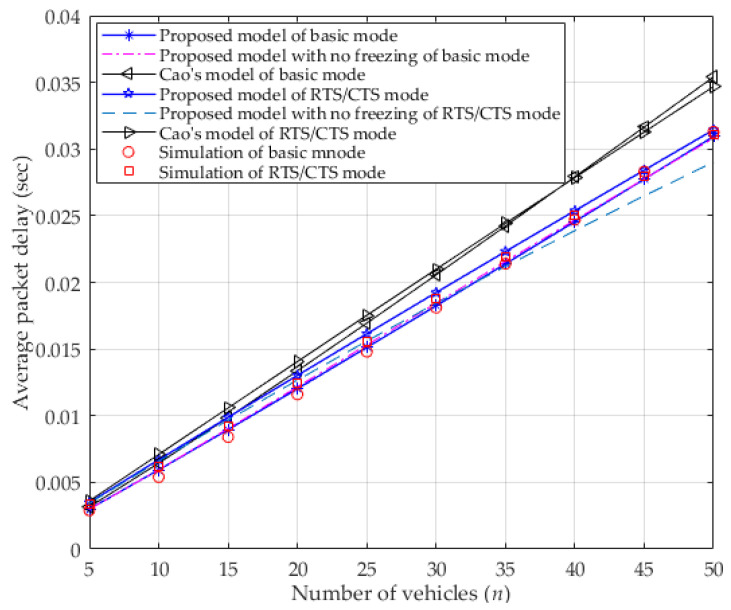
Average packet delay.

In Figure 6, the average packet delay in both modes is presented. As seen in the figure, when the number of vehicles increases, more vehicles contend for the channel, which enlarges the average packet delay. Since ignoring the freezing mechanism in the proposed model results in higher transmission probability (shown in Figure 4) can allow more chances for the vehicle to transmit in a period of time, and more collision will happen. Though more collisions lead to a higher probability of dropping packets (due to reaching the retry limit), the average packet delay is close to that which occurs when considering the freezing mechanism in the basic mode as its calculation excludes the time occupied by a packet that is dropped because it has reached the retry limit (as described in the first paragraph of Section 3.4). However, in RTS/CTS mode, the average packet delay of ignoring the freezing mechanism decreases as the number of vehicles increases, and more so than it does when considering the freezing mechanism. In fact, the vehicle can receive more transmission opportunities by using short control packets (i.e., RTS/CTS) when ignoring the freezing mechanism, which results in more successful transmission of data frames in a certain period of time, and accordingly, a decrease in the average packet delay. Nevertheless, the freezing mechanism is one of the key characteristics of the DCF that must be considered when analyzing the DCF’s performance. Moreover, the average packet delay of the proposed model is less than it is in Cao’s model in both two modes that consider the capture effect, but are consistent with the simulation result. Since the capture effect can increase the probability of successful transmission, the number of retransmissions decreases, as does the average packet delay. Moreover, the proposed model makes it is easy to find that the basic mode is more sensitive to the capture effect than it is to the RTS/CTS mode.

## 5. Conclusions

In this study, we propose a novel analytical model for analyzing the IEEE 802.11p DCF protocol that considers the capture effect under the Nakagami-*m* fading channel, which is different from the existing models and more suitable to the real VANET scenario. The comparisons between the simulations and the analytical results verify the accuracy of the proposed analytical model. The results show that the normalized saturated throughput that considers the capture effect is higher than it is without the capture effect. Moreover, the capture effect causes the average packet delay to decrease. Therefore, when analyzing IEEE 802.11p DCF (or EDCA) or designing the improved MAC protocols in VANETs, the capture effect under Nakagami-*m* fading channel is a noticeably valuable element that is worthy of consideration.

## Figures and Tables

**Table 1:** Notions used in the model.

Notation	Definition
$T_{E\left[PL\right]}$	Average length of Payload
$T_{H}$	Duration of header
$T_{ACK}$	Duration of ACK
$T_{RTS}$	Duration of RTS
$T_{CTS}$	Duration of CTS
$T_{SIFS}$	Duration of SIFS
$T_{DIFS}$	Duration of DIFS
$R_{t}$	Channel rate
m	The parameter of Nakagami fading
σ	Duration of a backoff slot
$T_{s}$	Average duration of a successful transmission
$T_{c}$	Average duration of a collided transmission
$T_{PD}$	Propagation delay
M	Maximum backoff stage
f	Retransmission times after reaching the maximum backoff stage
n	Number of nodes in the network
z	Capture threshold
$\gamma_{k}$	The signal power from node k
$\bar{\gamma }$	Average received power at each node
$p_{cap}$	Probability of capture effect
$p_{b}$	Probability that the channel is busy
$p_{c}$	Probability that the node observes a collided transmission
$W_{i}$	Contention window of backoff stage i
$W_{0}$	Minimum contention window
$W_{M}$	Maximum contention window
$\tau_{tra}$	Probability that a node transmits in a randomly chosen slot
$p_{tra}$	Probability that there is at least one transmission in the concerned slot
$p_{s}$	Probability that one node successfully transmits in a concerned slot conditioned on that at least one node transmits
E[Slot]	Average length of a virtual slot
η	Normalized throughput

**Table 2:** Simulation parameters.

Parameter	Setting	Parameter	Setting
E[PL]	512 bytes	$T_{SIFS}$	32 μs
$PHY_{hdr}$	224 bits	$T_{DIFS}$	58 μs
$MAC_{hdr}$	192 bits	σ	13 μs
ACK	304 bits	$T_{PD}$	1 μs
RTS	352 bits	$W_{0}$	32
CTS	304 bits	$W_{M}$	1024
$R_{t}$	11 Mbps	M	5
m	1.5	f	2
z	2	Simulation time	200 s

## Data Availability

Not applicable.
